# Impact of biliary fungal contamination on outcomes after pancreaticoduodenectomy for pancreatic cancer

**DOI:** 10.3389/fonc.2026.1776853

**Published:** 2026-02-27

**Authors:** Jeremy Chang, Sophia Xiao, Yutao Su, Scott K. Sherman, James R. Howe, James P. De Andrade, Hisakazu Hoshi, Carlos H. F. Chan

**Affiliations:** 1 Department of Surgery, University of Iowa Health Care, Iowa City, IA, United States; 2 Holden Comprehensive Cancer Center, University of Iowa Health Care, Iowa City, IA, United States

**Keywords:** biliary stenting, *Candida*, fungus, microbiome, neoadjuvant chemotherapy, pancreatic cancer, pancreaticoduodenectomy

## Abstract

**Introduction:**

Many patients with cancer of the pancreatic head will have biliary stenting to relieve malignant obstruction. Biliary stenting is associated with increased rates of bacterial and fungal biliary contamination. Little is known regarding the impact of fungal biliary contamination on postoperative and oncologic outcomes of pancreatic cancer. This study aims to evaluate the effects of fungal biliary contamination on postoperative and oncologic outcomes in patients receiving pancreatoduodenectomy for pancreatic ductal adenocarcinoma (PDAC).

**Methods:**

A retrospective study of a prospectively maintained single tertiary institutional database was performed, identifying patients with a diagnosis of PDAC from 2015 to 2022 who underwent curative-intent resection and had intraoperative biliary fungal cultures. Primary outcome measures assessed included overall survival (OS) and recurrence-free survival (RFS). The secondary outcome measure was postoperative complication rate. The Kaplan method estimated OS and RFS, and survival curves were compared with the log-rank test. Clinicopathologic variables were assessed for association with multivariable Cox hazard ratio.

**Results:**

Among 82 patients included, 87.8% had preoperative stenting. In stented patients, bacterial and fungal contamination had an incidence of 98.7% and 48.6%, respectively. Patients with positive fungal cultures had higher rates of neoadjuvant chemotherapy utilization than those with negative intraoperative fungal bile cultures (*p* = 0.05). Positive biliary fungal cultures were not independently associated with risk for postoperative complications nor RFS but were associated with worse OS (HR = 2.11 [1.04–4.26], *p* = 0.04). In the subgroup of patients who received neoadjuvant chemotherapy, positive fungal bile culture was associated with worse OS (HR = 2.70 [1.11–6.60], *p* = 0.03), but without more pronounced hematological evidence of systemic immunosuppression before and after chemotherapy.

**Conclusion:**

Biliary fungal contamination was not associated with increased risk of postoperative complications in patients with pancreatic cancer but was associated with worse OS, particularly in patients who received neoadjuvant therapy. Investigations regarding the causal relationship between biliary fungus and treatment response and outcome in patients with PDAC are warranted.

## 1 Introduction

Pancreatic ductal adenocarcinoma (PDAC) of the pancreatic head often presents at an advanced stage with malignant distal biliary obstruction (1). At the time of diagnosis, only approximately 10%–15% of patients are candidates for upfront curative resection (2). With increased adoption of neoadjuvant therapy (NAT) for borderline-resectable disease and ongoing trials for NAT in resectable disease, biliary drainage with stent placement is required to stabilize patients to receive NAT (3). However, biliary stenting has been demonstrated to be associated with increased rates of bacterial and fungal biliary contamination, with the literature reporting rates of 90% to 98% positive cultures after stenting (4–6). Contamination of the biliary tract causes bacterial and fungal dysbiosis, impacting the gut micro/mycobiome given its immediate proximity (7).

Duodenal dysbiosis and bacterial translocation is thought to not only impact postoperative outcomes but also directly alter PDAC tumor biology. From a surgical standpoint, early studies in pancreatectomy patients did not initially identify any difference in postoperative morbidity between patients who underwent preoperative stenting and those who did (8). More recently, positive intraoperative bile cultures have been associated with increased postoperative surgical infections (6, 9, 10). To mitigate this, a randomized trial found that broad-spectrum perioperative antibiotic coverage with piperacillin-tazobactam leads to decreased rates of infection and pancreatic leak compared with cefoxitin (11). The efficacy of piperacillin-tazobactam is due to the presence of cephalosporin-resistant bacterial species (12). Studies have implicated the gut microbiome in PDAC carcinogenesis through promotion of oncogenic signaling, chronic inflammation, immune interaction, and secretion of microbe-derived metabolites (13). While cancer–microbiome interactions and associations have been increasingly scrutinized, the role of the mycobiome remains understudied.

A 2022 study in *Cell* investigated fungal composition and diversity among 17,000 samples and 35 cancer types, finding increased expression of fungal signatures in tumor tissues as well as significant differences in fungal diversity across cancer types (14). Fungal species are thought to contribute to oncogenesis through activation of proinflammatory responses and generation of mutagenic metabolites (15). Conversely, fungal dysbiosis may also contribute to immunosenescence and immune exhaustion, impacting innate antitumor effects (16). However, the clinical operative morbidity and oncologic impact of fungal dysbiosis are poorly understood. A 2020 study by Tortajada et al. examined whether fungal biliary contamination in addition to bacterial contamination conferred additional risk for postoperative infection (as most patients had both) and found no additional postoperative risk (17). From an oncologic perspective, preliminary *in vitro* and *in vivo* studies have suggested that contaminated bile hinders host antitumor mechanisms, allowing for increased propagation of pancreatic cancer cells; however, these findings have not yet been supported from clinical data (18). This study seeks to evaluate the effects of fungal biliary contamination on postoperative and oncologic outcomes in patients receiving pancreatoduodenectomy for PDAC. We hypothesize that fungal biliary contamination is associated with increased rates of postoperative surgical site infections (SSIs) and pancreatic leaks as well as worse survival.

## 2 Methods

### 2.1 Study population

A retrospective study was performed at a single tertiary care institution from 2015 to 2022. Patients were included if they had (1) histopathologic diagnosis of PDAC, (2) cancer located in the head/neck/uncinate process of pancreas, (3) undergone curative intent resection with pancreatoduodenectomy, and (4) biliary culture data available for analysis. Patients were excluded if they had more than one lifetime cancer diagnosis or curative intent resection was aborted/unable to be completed. Patient demographic, oncologic, treatment, postoperative complications, and oncologic outcomes data were abstracted from their electronic medical records and the institutional American College of Surgeons National Surgical Quality Improvement Program (NSQIP) data. Biliary cultures were routinely obtained intraoperatively by the operating surgeons at the time of resection via needle aspiration of bile from the gallbladder prior to the cholecystectomy portion of pancreatoduodenectomy or if the patient had prior cholecystectomy via a culture swab of the common bile duct. All samples were sent for anaerobic and aerobic bacterial culture as well as fungal culture. Identification of bacterial species was performed by matrix-assisted laser desorption/ionization time-of-flight (MALDI-TOF) mass spectrometry (MS) in the University of Iowa Microbiology Lab, and culture results were reported semi-quantitatively for each identified species (rare, few, moderate, or many) in the electronic medical records. Primary study outcome measures were overall survival (OS) (calculated from the time of initial diagnosis) and recurrence-free survival (RFS) (calculated from the time of surgical resection). Secondary outcome measures were postoperative complication rates broken down into minor or major complications based on the Clavien–Dindo Classification, with specific attention to incidence of postoperative SSI and pancreatic leak. Pancreatic leak was defined by a postoperative day 3 amylase level greater than three times the upper limit of normal. The grade of postoperative pancreatic fistula was defined per International Study Group of Pancreas Surgery (ISGPS) criteria (19). This study was performed in compliance with the guidelines of the University of Iowa Human Subjects Office under an approved IRB protocol (IRB#202209470).

### 2.2 Statistical analysis

Summary statistics were used to describe the study population. Categorical variables were compared with the chi square test or Fisher’s exact test, and continuous variables were compared with the Mann–Whitney *U* test. The Kaplan–Meier method estimated OS and RFS. Kaplan–Meier survival curves were compared for significance with the log-rank test. Univariate and multivariate Cox hazard ratio analysis was utilized to identify variables associated with a positive or negative effect on OS or RFS. Univariable and multivariable regression was utilized to investigate the association of variables with minor and major complications, SSI, and pancreatic leak. Only variables significant on univariable regression were included in multivariable analysis. Demographic, cancer, pancreas, and treatment variables were included in multivariate analysis to account for confounding variables. Analyses were performed with R via R Studio (20).

## 3 Results

### 3.1 Study population

**Figure 1:**
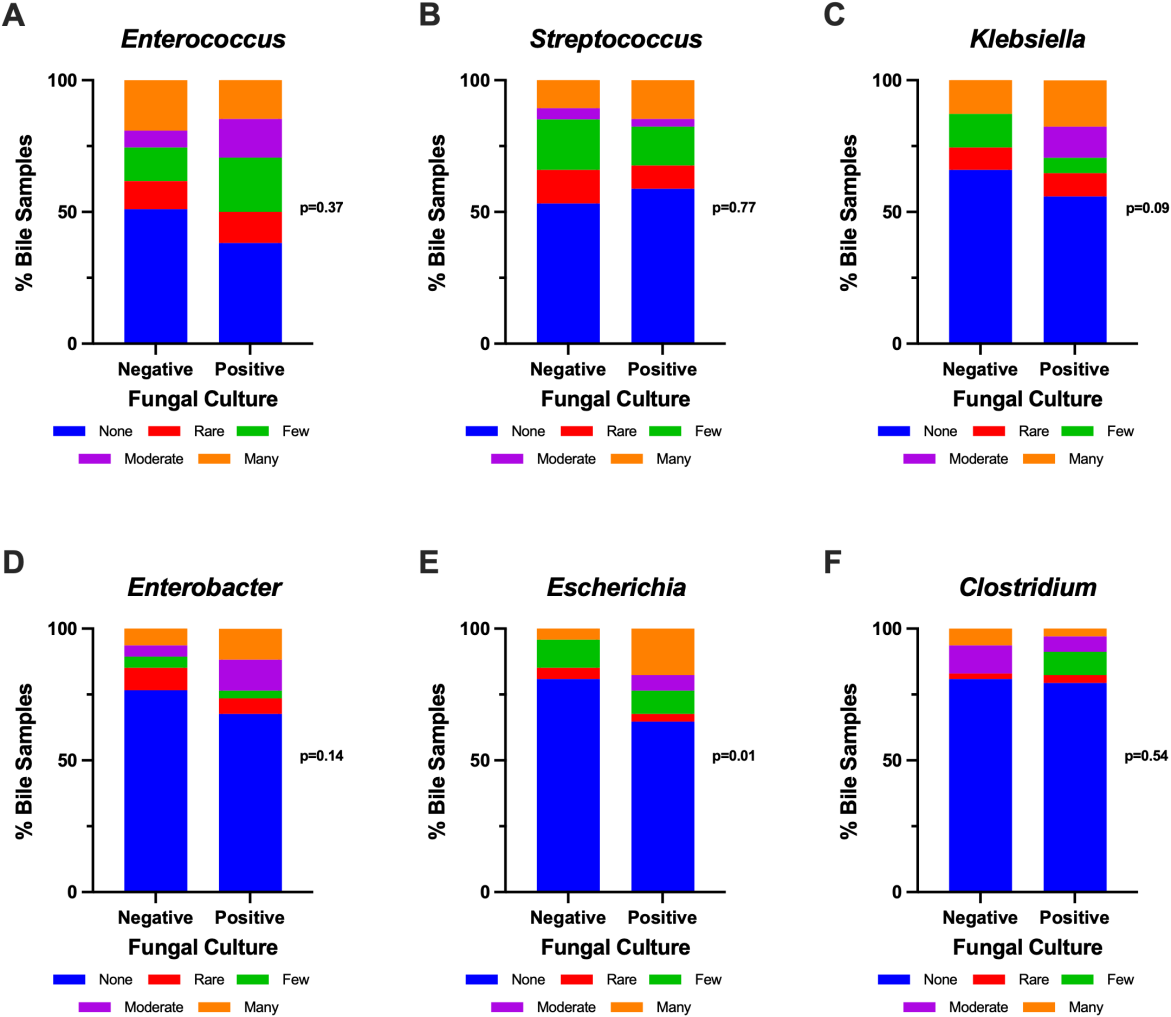
Association between commonly isolated bacterial species and fungal positivity in intraoperative bile cultures: *Enterococcus* spp. **(A)**, *Streptococcus* spp. **(B)**, *Klebsiella* spp. **(C)**, *Enterobacter* spp. **(D)**, *Escherichia coli***(E)**, and *Clostridium* spp. **(F)**. Bacterial species were identified by MALDI-TOF MS in the University of Iowa Microbiology Lab and culture results were reported semi-quantitatively for each identified species (rare, few, moderate, or many). Statistical analyses were performed using chi-square tests.

A total of 82 patients with PDAC of the head/neck/uncinate process of the pancreas who received curative intent pancreatoduodenectomy were included. In addition, nine (11.0%) patients received irreversible electroporation (IRE). The rate of preoperative stenting was 87.8%, and the rate of receipt of NAT was 68.3% (68.3% with neoadjuvant chemotherapy and 41.5% with neoadjuvant radiation). All patients received perioperative prophylactic antibiotics without antifungal drugs, and a total of 17 (20.7%) patients received antifungal treatment—2 prior to surgery and 15 within 30 days after surgery for documented fungal infections. Of patients who underwent preoperative stenting, the rate of bacterial biliary contamination and fungal biliary contamination was 98.7% and 48.6%, respectively. All patients with positive fungal bile culture had positive bacterial bile cultures as well. All but 1 of the 35 patients with positive fungal bile cultures were colonized with *Candida* (one patient had *Saccharomyces cerevisiae*), with the most common subspecies being *C. albicans* (64.7%) and *C. glabrata* (20.6%). *Enterococcus* (54.2%), *Streptococcus* (44.4%), *Klebsiella* (38.3%), *Enterobacter* (27.2%), *Escherichia* (25.9%), *Prevotella* (22.0%), and *Clostridium* (20.7%) were the common bacterial species found in bile. While there were no significant correlations between fungal culture positivity and most of these bacterial genus or species (**Figure 1**), fungal-positive bile samples tended to contain more *Klebsiella* species (44.1% vs. 34.0%, *p* = 0.09) and significantly more *Escherichia coli* (35.3% vs. 19.2%, *p* = 0.01).

There was no significant difference in age, sex, American Society of Anesthesia (ASA) class, or pathologic cancer staging between patients with positive fungal bile cultures and those without (**Table 1**). The pancreas tended to have a larger duct diameter (25.7% of duct >6 mm vs. 8.5%, *p* = 0.12) and a firmer gland texture (5.7% with soft gland vs. 25.5%, *p* = 0.04) in patients with positive fungal cultures. Patients with positive fungal cultures had higher rates of preoperative biliary stenting (100% vs. 78.7%, *p* = 0.01), neoadjuvant chemotherapy (80.0% vs. 59.6%, *p* = 0.05), and neoadjuvant chemoradiation (54.3% vs. 31.9%, *p* = 0.04) than those with negative intraoperative fungal bile cultures.

**Table 1:** Study population.

Variables	Positive fungal cultures (*n* = 35)	Negative fungal cultures (*n* = 47)	*p*-value
Age (median [IQR])	66.5 [61.1–73.8]	69.5 [61.0–73.4]	0.71
Gender			0.59
Male	17 (48.6%)	20 (42.6%)	
Female	18 (51.4%)	27 (57.4%)	
Race			0.42
White	32 (91.4%)	45 (95.7%)	
African American	3 (8.6%)	2 (4.3%)	
ASA Class			0.68
2	13 (37.1%)	22 (46.8%)	
3	21 (60.0%)	24 (51.1%)	
4	1 (2.9%)	1 (2.1%)	
Preoperative stenting	35 (100.0%)	37 (78.7%)	**0.01**
Neoadjuvant chemo	28 (80.0%)	28 (59.6%)	**0.05**
Neoadjuvant chemoradiation	19 (54.3%)	15 (31.9%)	**0.04**
Received IRE	5 (14.3%)	4 (8.5%)	0.41
Received anti-fungal drug	8 (22.9%)	9 (19.1%)	0.79
Patdological stage			0.66
IA	4 (11.4%)	9 (19.1%)	
IB	3 (8.6%)	5 (10.6%)	
IIA	2 (5.7%)	0	
IIB	24 (68.6%)	30 (63.8%)	
Unknown	2	3	
Pancreas duct size			0.12
<3 mm	17 (48.6%)	29 (61.7%)	
3–6 mm	9 (25.7%)	12 (25.5%)	
>6 mm	9 (25.7%)	4 (8.5%)	
Unknown	0	2	
Gland texture			**0.04**
Soft	2 (5.7%)	12 (25.5%)	
Intermediate	20 (57.1%)	18 (38.3%)	
Hard	13 (37.1%)	15 (31.9%)	
Unknown	0	2	

Bold values denote p-value < 0.05.

### 3.2 Postoperative outcomes

The overall rate of any postoperative complication for the entire cohort was 39.0% with 19 (23.2%) patients having minor complications (i.e., Clavien–Dindo grade 1 or 2) and 13 (15.8%) patients having major complication (i.e. Clavien–Dindo grade 3 or 4). The rate of any SSI was 25.6% divided into 10 (12.2%) patients with superficial SSI and 11 (13.4%) patients with organ space SSI. The rate of any pancreatic leak was 12.2% divided into 6 (7.3%) patients with biochemical leaks and 4 (4.9%) patients with grade B leaks. There was no case of grade C leak. Fifteen patients (18.3%) had unplanned readmissions within 30 days of procedure. There was no significant difference in overall complication rate, SSI rate, or pancreatic leak rate between patients with positive intraoperative fungal cultures and those with negative intraoperative fungal cultures (**Table 2**).

**Table 2:** Comparison of complication rates.

Variables	Positive fungal cultures (*n* = 35)	Negative fungal cultures (*n* = 47)	*p*-value
Any complication	15 (42.9%)	17 (36.2%)	0.54
Clavien–Dindo 1/2	12 (34.3%)	7 (14.9%)	**0.04**
Clavien–Dindo 3/4	3 (8.6%)	10 (21.3%)	0.12
Any SSI	12 (34.3%)	9 (19.1%)	0.11
Superficial SSI	6 (17.1%)	4 (8.5%)	0.24
Organ space SSI	6 (17.1%)	5 (10.6%)	0.39
Any pancreatic leak	3 (8.6%)	7 (14.9%)	0.39
Biochemical leak	1 (2.9%)	5 (10.6%)	0.18
Grade B/C	2 (5.7%)	2 (4.3%)	0.76
Readmission within 30 days	7 (20.0%)	8 (17.0%)	0.73

Bold values denote p-value < 0.05.

Univariate and multivariable regression was performed to identify risk factors for SSI and pancreatic leak. Regression models for SSI and pancreatic leak included the following variables: age, sex, ASA class, receipt of neoadjuvant chemotherapy, receipt of neoadjuvant chemoradiation, fungal bile culture result, bacterial bile culture result, stage, pancreatic duct size, and pancreatic gland texture. There was no independent risk factor identified impacting SSI occurrence (**Supplementary Table 1**) or pancreatic leak (**Supplementary Table 2**). Positive intraoperative fungal bile cultures did not appear to be a risk factor for SSI or pancreatic leak occurrence in this study cohort.

### 3.3 Oncologic outcomes

The median OS for the study cohort was 48 [32–Not Reached (NR)] months, and the median RFS was 34 [24–NR] months. The median follow-up for the study cohort was 35 [31–40] months. Total number of death and recurrence events were 34 and 28, respectively. Kaplan–Meier survival curves were generated for OS and RFS comparing cohorts with positive versus negative intraoperative fungal biliary cultures, and there was a significant difference in estimated OS, but not RFS (**Supplementary Figure 1**). Univariable and multivariable Cox hazard ratio analysis was utilized to assess for variables affecting OS or RFS. In a model for OS including age, sex, ASA classification, receipt of neoadjuvant chemotherapy, receipt of neoadjuvant chemoradiation, pathologic cancer stage, fungal bile culture result, bacterial bile culture result, receipt of antifungal drug, and occurrence of complication, both ASA class 4 (HR 5.70 [1.23–26.41], *p* = 0.03) and positive fungal culture (HR 2.11 [1.04–4.26], *p* = 0.04) were independent risk factors for worse OS (**Supplementary Table 3**). In a model for RFS including age, sex, receipt of neoadjuvant chemotherapy, pathologic cancer stage, fungal bile culture result, bacterial bile culture result, and receipt of antifungal drug, only Stage II disease (relative to Stage I) was an independent risk factor (HR = 3.74 [1.13–12.36], *p* = 0.03) for worse RFS (**Supplementary Table 4**).

**Figure 2:**
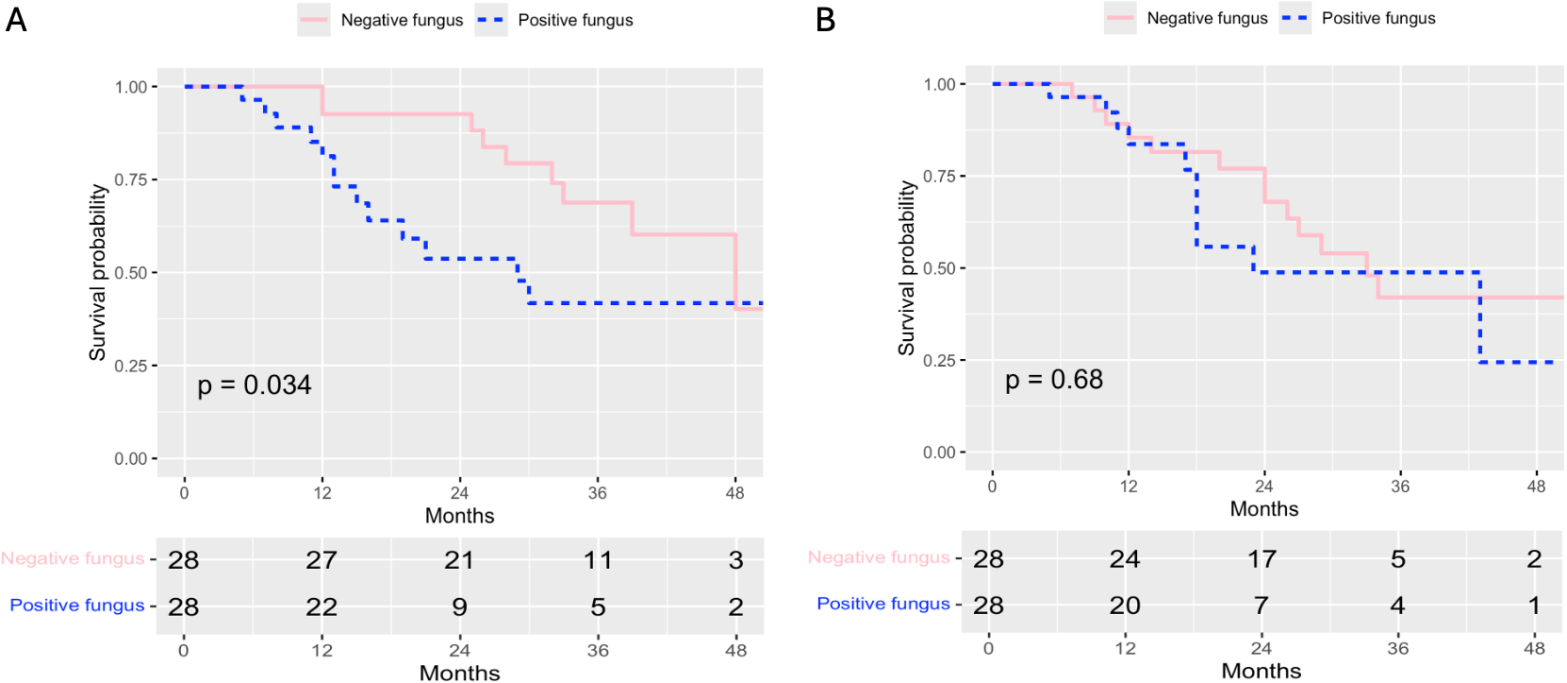
Kaplan–Meier curves for **(A)** OS and **(B)** RFS in patients receiving neoadjuvant chemotherapy with positive and negative fungal bile cultures. Kaplan–Meier curves were compared with log-rank test for alpha significance. **(A)** Survival curves for OS. Median overall survival was 29 months (95% CI: 16–Not reached) for positive bile fungus vs. 48 months (95% CI: 39–Not reached) for negative bile fungus, *p* = 0.034. *N* = 28 for negative fungus with 9 death events. *N* = 28 for positive fungus with 14 death events. **(B)** Survival curves for RFS. Median recurrence-free survival was 23 months (95% CI: 18–Not reached) for positive bile fungus vs. 33 months (95% CI: 26–Not reached) for negative bile fungus, *p* = 0.68. *N* = 28 for negative fungus with 13 recurrence events. *N* = 28 for positive fungus with 10 recurrence events.

Additional subgroup analyses were performed investigating OS and RFS in patients who had received neoadjuvant chemotherapy or upfront resection. Comparison of Kaplan–Meier survival curves of OS and RFS between patients receiving neoadjuvant chemotherapy with positive and negative fungal biliary cultures demonstrated worse OS (29 vs. 48 months, *p* = 0.03) and no difference in RFS for patients with positive fungal bile cultures (**Figure 2**). In multivariable analysis for OS including only patients receiving NAT, both ASA class 4 (HR = 9.51 [1.78–50.97], *p* = 0.01) and positive fungal bile cultures (HR = 2.70 [1.11–6.60], *p* = 0.03) were independent risk factors for worse OS (**Table 3**). In RFS analysis, Stage II disease remained an independent risk factor for worse RFS (**Supplementary Table 5**). Among patients who received upfront resection, there was no association between positive fungal bile culture and OS or RFS (**Supplementary Tables 6** and **7**).

**Table 3:** Univariable and multivariable Cox hazard ratio analysis for risk factors for overall survival in patients who received neoadjuvant therapy.

Variables	Univariable		Multivariable	
	HR [95% CI]	*p*-value	HR [95% CI]	*p*-value
Age >65 (Ref: age <65)	0.82 [0.33–2.05]	0.68		
Male sex (Ref: female)	0.80 [0.35–1.85]	0.60		
ASA class (Ref: Class 2)				
Class 3	2.30 [0.88–5.99]	0.09	2.12 [0.81–5.55]	0.19
Class 4	**6.47 [1.28**–**32.60]**	**0.03**	**9.51 [1.78**–**50.97]**	**0.01**
Stage II (Ref: Stage I)	1.98 [0.73–5.37]	0.18		
Positive fungal culture (Ref: negative culture)	**2.43 [1.04**–**5.66]**	**0.04**	**2.70 [1.11**–**6.60]**	**0.03**
Positive bacterial culture (Ref: negative culture)	1.20 [0.28–5.20]	0.80		
*Enterococcus* spp.	0.84 [0.37–1.94]	0.69		
*Enterobacter* spp.	1.24 [0.52–2.94]	0.63		
*Klebsiella* spp.	1.16 [0.51–2.66]	0.72		
*Streptococcus* spp.	0.64 [0.27–1.52]	0.31		
*Escherichia* spp.	1.36 [0.49–3.80]	0.56		
*Prevotella* spp.	0.49 [0.17–1.46]	0.20		
*Clostridium* spp.	1.33e-8 [0–inf]	1.00		
Received anti-fungal drug	1.50 [0.55–4.08]	0.43		
Any complication occurrence (Ref: no complication)	1.29 [0.55–3.02]	0.56		

Bold values denote p-value < 0.05.

Positive fungal bile culture appeared to be a negative risk factor for OS, specifically in patients receiving neoadjuvant chemotherapy. To investigate whether this was associated with response to neoadjuvant chemotherapy, we compared pathologic tumor regression grade (TRG) and CA19–9 normalization. TRG in patients with positive fungal bile culture was numerically worse, but this difference was not statistically significant (TRG 3: 22.2% vs. 7.1%, *p* = 0.14). Although not statistically significant, 4/28 (14.8%) of patients with positive fungal culture had positive resection margin (R1 resection), while in those with negative fungal culture, 1/28 (3.6%) had R1 resection (*p* = 0.16). Post-NAT pathologic perineural invasion rate was higher in those with positive fungal culture (75% vs. 50%, *p* = 0.05); however, there was no difference in post-NAT lymphovascular invasion (53.5% vs. 39.3%, *p* = 0.28). Patients with positive fungal culture after NAT had a lower biomarker response numerically but did not reach statistical significance (CA19–9 normalized in 27.8% vs. 50.0%, *p* = 0.29; mean CA19–9 reduction of 59.9% vs. 71.6%, *p* = 0.27).

**Figure 3:**
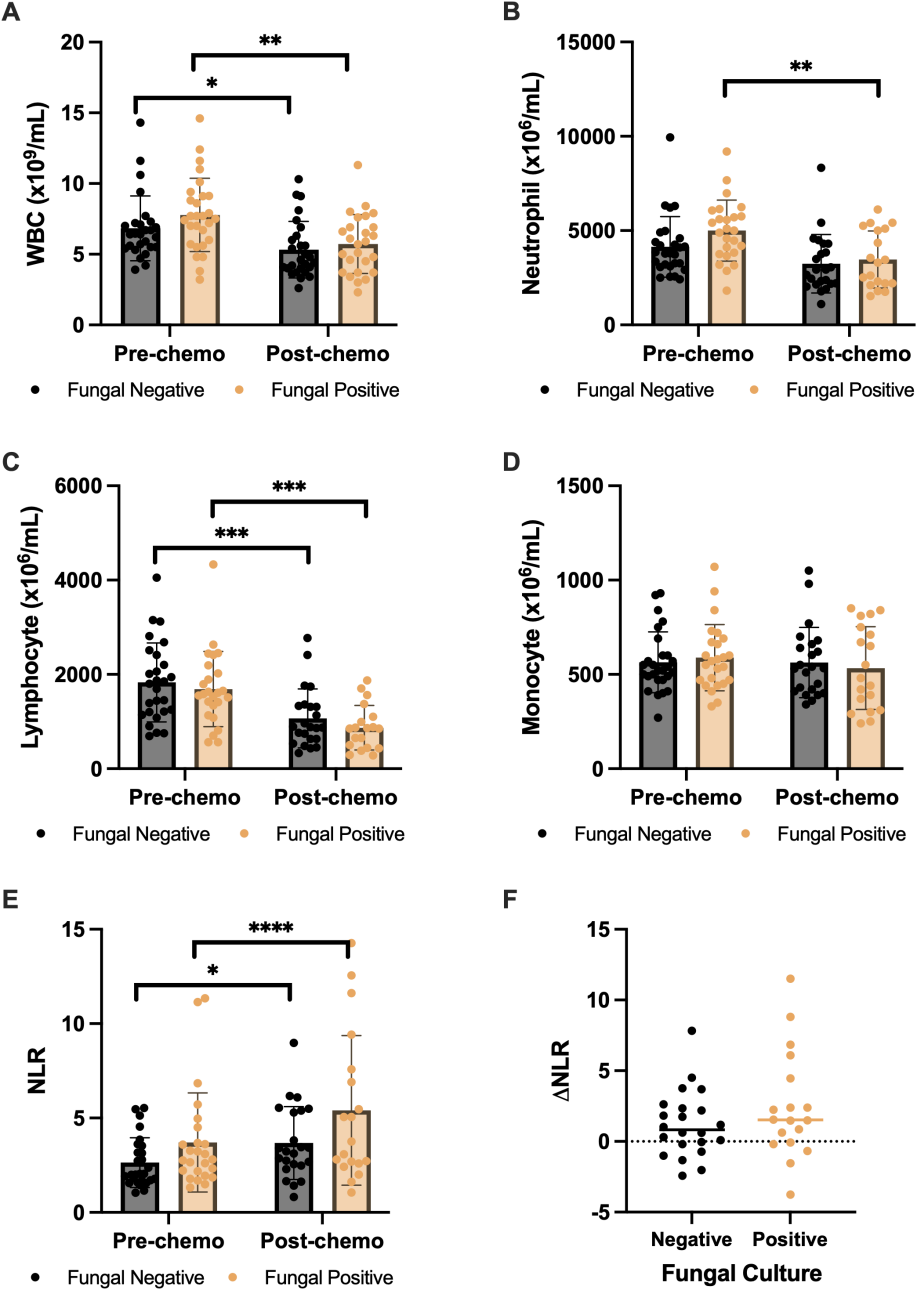
Circulating immune cell counts in patients with PDAC before and after neoadjuvant chemotherapy: WBC **(A)**, neutrophil **(B)**, lymphocyte **(C)**, monocyte **(D)**, NLR **(E)**, and ΔNLR **(F)**. Statistical analyses were performed using Student’s *t*-tests. No significant difference was detected between fungal-negative and fungal-positive groups. * p-value < 0.05; ** p-value < 0.01; *** p-value < 0.001; **** p-value < 0.0001.

To investigate whether the presence of biliary fungus after NAT was associated with systemic immunosuppression that could have an impact on OS, circulating immune cell differentials before and after neoadjuvant chemotherapy between patients with and without positive fungal culture were compared. There was no significant difference in immune cell counts between patients with and without positive fungal culture in pre-chemotherapy or post-chemotherapy blood samples (**Figure 3**). Overall, there were significant decreases after chemotherapy in the fungal-negative group in mean white blood count (WBC; 6.84 × 10^9^/mL vs. 5.32 × 10^9^/mL, *p* = 0.01), mean neutrophil count (4.14 × 10^6^/mL vs. 3.24 × 10^6^/mL, *p* = 0.05), and mean lymphocyte count (1.83 × 10^6^/mL vs. 1.07 × 10^6^/mL, *p* = 0.001) and in the fungal-positive group in mean WBC (7.78 × 10^9^/mL vs. 5.71 × 10^9^/mL, *p* = 0.002), mean neutrophil count (5.00 × 10^6^/mL vs. 3.47 × 10^6^/mL, *p* = 0.003), and mean lymphocyte count (1.69 × 10^6^/mL vs. 0.87 × 10^6^/mL, *p* = 0.0001). When comparing neutrophil-to-lymphocyte ratio (NLR), there were significant increases after chemotherapy in the fungal-negative group (2.6 vs. 3.7, *p* = 0.035) and the fungal-positive group (3.7 vs. 5.4, *p* < 0.0001). While the fungal-positive group had higher NLRs in both pre-chemotherapy and post-chemotherapy, there was no significant difference when doing a pairwise analysis (**Figure 3F**).

## 4 Discussion

This study identifies 82 patients with PDAC of the pancreatic head who underwent curative-intent resection and had intraoperative biliary cultures performed. The rate of fungal bile contamination in patients who underwent preoperative biliary decompression was 48.6%, which is somewhat higher than the rate reported in a previous study (17). Contrary to our hypothesis, we found that positive intraoperative fungal cultures were not significantly associated with immediate postoperative morbidity. However, we found that positive fungal bile culture was an independent risk factor of worse OS, particularly in the subgroup of patients who received neoadjuvant chemotherapy. Interestingly, among patients who received NAT, there was no difference in RFS between patients with positive fungal cultures and those without, although patients with positive fungal culture tended to have higher odds of R1 resection margin and perineural invasion.

Alterations in the gut mycobiome and the rise of certain “pathogenic” fungi have been linked to cancer tumorigenesis, first in colon and liver cancer but more recently in pancreatic cancer (21–23). Aykut et al. reported a >3,000-fold increase in fungal populations in PDAC tumor tissues compared to normal pancreas. While their proposed mechanism for the mycobiome dysbiosis in pancreatic tumors is translocation of endoluminal gut microbes from the sphincter of Oddi, it was unclear if the fungal colonization is promoted or accelerated by the introduction of microbes through endoscopic biliary procedures and biliary foreign bodies. They found that PDAC progression could be linked to fungal dysbiosis, specifically a rise in *Malassezia* spp., and that ablation of these species with antifungal treatment or reintroduction of *Malassezia* spp. directly correlated with oncogenesis (24). In the study by Aykut et al., they found that reintroduction of *Candida* spp. in antifungal-treated mice did not have the impact on oncogenesis that *Malassezia* spp. did in their *in vivo* model. *Malassezia* has been shown to play a role in PDAC oncogenesis through complement activation through mannose binding lectin (MBL) and effects on the immune microenvironment (24–26). These data, however, have not been comprehensively validated and generalized to humans. Analysis of 18S rRNA (fungal) sequencing samples from both normal pancreas and PDAC tumors did not identify any significant differences in mycobiome composition; however, this could likely be due to very low sequencing reads in the tissue samples (27). In this study, we did not identify the presence of any *Malassezia* spp. in the biliary cultures, and contamination with *Candida* spp. was the most common. Recently, a 2025 study by Meng et al. sequenced the oral wash of 445 patients who developed pancreatic cancer matched with control epidemiological cohorts and found that increased abundance of *Candida* was associated with increased risk for pancreatic cancer (28). Additionally, fungus in PDAC has been suggested to interact with the tumor microenvironment through induction of interleukin 33 (IL-33) expression, which creates a proinflammatory response, increasing local density of T helper 2 (Th2) and T-regulatory (Treg) cells. This is associated with tumor proliferation (26).

The current study finds that positive fungal bile cultures negatively impact OS, but not RFS in patients with PDAC who received neoadjuvant chemotherapy. We hypothesize that a possible reason for this phenomenon is decreased responsiveness to neoadjuvant chemotherapy, but the exact mechanism for this is currently unknown (29). Another explanation is that there is some correlation between positive bile fungus and the patient’s systemic condition as ASA class 4 was a significant predictor of worse OS. It has been previously demonstrated that patients with PDAC who receive NAT have a proinflammatory immune microenvironment that is associated with antitumor effects (30–32). These microenvironment changes in patients who have improved survival with NAT is characterized by expansion of T-cell populations, specifically CD8 cytotoxic T cells, and decreases in immunosuppressive M2 macrophage and granulocyte populations (31). Whether dysbiosis of the PDAC microenvironment can specifically affect NAT response is yet to be determined. It has been suggested that fungal dysbiosis may increase Th17 populations, leading to immunosuppression and decreased antitumor activity (33, 34). Fungi themselves may additionally have multifaceted effects. *C. albicans* colonization in mice has been shown to increase the proportion of immunosuppressive PD-1^+^CD8^+^ T cells in colon cancer (35). In *C. albicans*-dominant gastric cancers, there is significant upregulation of proinflammatory genes (36). In pancreatic cancer, Ear et al. have recently shown that the presence of tumor-associated *C. albicans* is associated with an epithelial–mesenchymal transition (EMT) gene signature in PDAC (26, 37). EMT is a well-described regulator in invasive PDAC progression as well as drug resistance (38–40).

There has been increased research into the PDAC immune microenvironment changes associated with PDAC fungal dysbiosis. One goal of these studies is to determine if regulation of an “oncologic” mycobiome with antifungal medications could be a new avenue of treatment. Preliminary *in vivo* mouse studies have shown that antifungal treatment can lead to decreased PDAC tumorigenesis and aggressiveness (24, 26, 41). Treatment of mice with the antifungal B-glucan induces a transition of immunosuppressive M2 macrophages to proinflammatory M1 macrophages, and increases NK cell cytotoxicity, increasing innate antitumor effects (42–44). A recent *in vivo* study combining the antifungal itraconazole with anti-PD1/-CTLA4 immunotherapy demonstrated improvements in survival in mouse models (45). While antifungal treatment was not shown to correlate with survival outcomes in our study, we could not make a definitive conclusion since only 20% of our study patients received a short course antifungal treatment for perioperative infections. The impact of long-term suppressive antifungal treatment on chemotherapy response and survival of patients with PDAC has not been evaluated in a human clinical trial setting.

This study is limited by the size of the study population and the use of semi-quantitative clinical microbiology data without quantitative metagenomic data analysis. Although 56 (68.3%) patients in the study received NAT, only 42 patients had pre- and post-NAT complete blood count with differential available to review limiting our ability to identify significant immunological differences between groups. The interpretation of systemic immunological state is also limited by the lack of cell counts of specific lymphocyte subsets, such as CD4/CD8 T lymphocytes, and systemic immunosuppression could only be defined by an absolute lymphocyte count of <1,000/mL or an absolute neutrophil count of <1,500/mL. Additionally, we are limited by the retrospective observational review nature of the study, including accounting for the presence of missing data as well as the inability to establish causality. The inability to abstract cause of death and the potential omission of some recurrence events could have impacts on the interpretation of OS and RFS analysis. While most patients with PDAC often die from their recurrent diseases based on our clinical experience, we are unable to estimate cancer-related OS and show that it is worse in fungal-positive patients with PDAC. Further larger-scale retrospective or prospective studies are warranted to further evaluate the impact of biliary contamination on NAT effectiveness. The causal relationship between biliary fungus and treatment response and outcome in patients with PDAC remains to be elucidated. While emerging evidence suggests that tumor-associated fungus may have direct impact on the tumor microenvironment and carcinogenesis, the impact of fungal colonization on immune modulation remains theoretical based on previous literature and is not proven in this clinical study.

In summary, this study finds that effectively all cases of PDAC fungal biliary contamination are associated with preoperative stenting. While positive fungal bile cultures do not seem to have an impact on postoperative complications, they are associated with worse OS in patients who received NAT without more pronounced hematological evidence of systemic immunosuppression. These clinical data support previous literature that fungal dysbiosis can impact the tumor microenvironment and the immune cell microenvironment in PDAC, and antifungal treatment may have an impact on cancer outcomes. These results establish that investigation of a relationship between NAT and biliary fungus may help to shed light on the mechanism by which PDAC biology alters therapy response.

## Data Availability

The original contributions presented in the study are included in the article/**Supplementary Material**. Further inquiries can be directed to the corresponding author.
