# Supplementary material for: Impact of biliary fungal contamination on outcomes after pancreaticoduodenectomy for pancreatic cancer
Source: Front Oncol. 2026 Feb 27;16:1776853. doi: 10.3389/fonc.2026.1776853 (PMC12982062; doi:10.3389/fonc.2026.1776853)
Supplement: Supplementary file 1 [file DataSheet1.docx]

Supplementary Material

# Supplementary Tables

**Supplemental Table 1.** Univariable Odds Ratio Analysis for Risk Factors for Surgical Site Infection

|  | Univariable | |
| --- | --- | --- |
|  | OR [95% CI] | p-value |
| Positive Fungal Culture | 1.16 [0.96 - 1.41] | 0.12 |
| Positive Bacterial Culture | 1.07 [0.80 - 1.43] | 0.67 |
| *Enterococcus spp.* | 1.14 [0.95 - 1.38] | 0.17 |
| *Streptococcus spp.* | 1.01 [0.84 - 1.23] | 0.88 |
| *Klebsiella spp.* | 1.13 [0.93 - 1.38] | 0.23 |
| *Enterobacter spp.* | 0.95 [0.77 - 1.17] | 0.61 |
| *Escherichia spp.* | 1.25 [0.99 - 1.59] | 0.07 |
| *Prevotella spp.* | 0.90 [0.72 - 1.14] | 0.40 |
| *Clostridium spp.* | 1.04 [0.80 - 1.34] | 0.78 |
| Age>65 | 1.12 [0.92 - 1.36] | 0.27 |
| Neoadjuvant Chemotherapy | 0.93 [0.76 - 1.14] | 0.42 |
| Neoadjuvant Chemoradiation | 1.07 [0.88 - 1.30] | 0.51 |
| Stage II (ref: stage 1) | 1.23 [0.98 - 1.56] | 0.08 |
| Pancreatic Duct Size (ref <3mm) |  |  |
| 3-6mm | 0.98 [0.78 - 1.23] | 0.85 |
| >6mm | 1.05 [0.80 - 1.38] | 0.74 |
| Pancreatic Gland Texture (ref: firm) |  |  |
| soft | 1.00 [0.75 - 1.33] | 1.00 |
| intermediate | 0.95 [0.77 - 1.18] | 0.66 |

**Supplemental Table 2.** Univariable and Multivariable Odds Ratio Analysis for Risk Factors for Pancreatic Leak (only includes Grade B/C leak).

|  | Univariable | |
| --- | --- | --- |
|  | OR [95% CI] | p-value |
| Positive Fungal Culture | 1.01 [0.92 - 1.12] | 0.77 |
| Positive Bacterial Culture | 0.94 [0.82 - 1.09] | 0.43 |
| *Enterococcus spp.* | 0.95 [0.87 - 1.05] | 0.36 |
| *Streptococcus spp.* | 1.07 [0.97 - 1.18] | 0.17 |
| *Klebsiella spp.* | 0.98 [0.88 - 1.08] | 0.63 |
| *Enterobacter spp.* | 1.05 [0.95 - 1.17] | 0.32 |
| *Escherichia spp.* | 1.02 [0.90 - 1.15] | 0.78 |
| *Prevotella spp.* | 1.09 [0.97 -1.22] | 0.14 |
| *Clostridium spp.* | 1.03 [0.91 - 1.16] | 0.67 |
| Age>65 | 0.97 [0.88 - 1.06] | 0.49 |
| Neoadjuvant Chemotherapy | 0.96 [0.87 - 1.06] | 0.43 |
| Neoadjuvant Chemoradiation | 0.92 [0.84 - 1.01] | 0.09 |
| Stage II (ref: stage 1) | 1.00 [0.99 -1.12] | 0.94 |
| Pancreatic Duct Size (ref <3mm) |  |  |
| 3-6mm | 0.94 [0.84 - 1.05] | 0.26 |
| >6mm | 1.01 [0.88 - 1.16] | 0.87 |
| Pancreatic Gland Texture (ref: firm) |  |  |
| soft | 1.04 [0.90 - 1.19] | 0.62 |
| intermediate | 1.02 [0.91 - 1.13] | 0.76 |

**Supplementary Table 3. Univariable and Multivariable Cox Hazard Ratio Analysis for Risk Factors for Overall Survival**

|  | Univariable | | Multivariable | |
| --- | --- | --- | --- | --- |
|  | HR [95% CI] | p-value | HR [95% CI] | p-value |
| Age >65 (Ref: Age <65) | 1.16 [0.56 - 2.40] | 0.69 |  |  |
| Male Sex (Ref: Female) | 0.75 [0.37 - 1.52] | 0.42 |  |  |
| ASA Class (Ref: Class 2) |  |  |  |  |
| Class 3 | 1.51 [0.72 - 3.19] | 0.28 | 1.42 [0.67 - 3.01] | 0.35 |
| Class 4 | **4.74 [1.04 - 21.59]** | **0.04** | **5.70 [1.23 - 26.41]** | **0.03** |
| Neoadjuvant Chemotherapy | 1.08 [0.51 - 2.28] | 0.85 |  |  |
| Neoadjuvant Chemoradiation | 1.18 [0.60 - 2.35] | 0.63 |  |  |
| Stage II (Ref: Stage I) | 1.93 [0.74 - 5.02] | 0.18 |  |  |
| Positive Fungal Culture (Ref: Negative Culture | **2.03 [1.02 - 4.05]** | **0.04** | **2.11 [1.04 - 4.26]** | **0.04** |
| Positive Bacterial Culture (Ref: Negative Culture) | 1.36 [0.48 - 3.90] | 0.56 |  |  |
| *Enterococcus spp.* | 0.82 [0.41 - 1.63] | 0.57 |  |  |
| *Streptococcus spp.* | 0.84 [0.42 - 1.70] | 0.63 |  |  |
| *Klebsiella spp.* | 1.35 [0.67 - 2.73] | 0.40 |  |  |
| *Enterobacter spp.* | 0.90 [0.42 - 1.94] | 0.79 |  |  |
| *Escherichia spp.* | 1.28 [0.51 - 3.19] | 0.60 |  |  |
| *Prevotella spp.* | 0.46 [0.16 - 1.31] | 0.15 |  |  |
| *Clostridium spp.* | 0.65 [0.20 - 2.14] | 0.48 |  |  |
| Received Anti-Fungal Drug | 1.71 [0.76 - 3.85] | 0.20 |  |  |
| Any Complication Occurrence (Ref: No Complication) | 1.44 [0.73 - 2.87] | 0.30 |  |  |

**Supplementary Table 4. Univariable Analysis for Risk Factors for Recurrence Free Survival**

|  | Univariable | |
| --- | --- | --- |
|  | HR [95% CI] | p-value |
| Age >65 (Ref: Age <65) | 0.48 [0.21 - 1.13] | 0.09 |
| Male Sex (Ref: Female) | 0.99 [0.49 - 2.02] | 0.99 |
| ASA Class (Ref: Class 2) |  |  |
| Class 3 | 0.95 [0.47 - 1.94 | 0.99 |
| Class 4 | 3.87e-0.8 [0.00 - inf] | 1.00 |
| Neoadjuvant Chemotherapy | 1.27 [0.57 - 2.85] | 0.56 |
| Neoadjuvant Chemoradiation | 0.99 [0.49 - 2.02] | 0.98 |
| Stage II (Ref: Stage I) | **3.74 [1.13 - 12.36]** | **0.03** |
| Positive Fungal Culture (Ref: Negative Culture | 1.33 [0.64 - 2.75] | 0.45 |
| Positive Bacterial Culture (Ref: Negative Culture) | 0.75 [0.31 - 1.85] | 0.54 |
| *Enterococcus spp.* | 0.67 [0.33 - 1.36] | 0.27 |
| *Streptococcus spp.* | 0.85 [0.41 - 1.76] | 0.66 |
| *Klebsiella spp.* | 0.60 [0.27 - 1.34] | 0.22 |
| *Enterobacter spp.* | 0.69 [0.30 - 1.61] | 0.39 |
| *Escherichia spp.* | 1.29 [0.52 - 3.18] | 0.59 |
| *Prevotella spp.* | 0.78 [0.32 - 1.91] | 0.59 |
| *Clostridium spp.* | 0.69 [0.21 - 2.29] | 0.56 |
| Received Anti-Fungal Drug | 1.04 [0.42 - 2.55] | 0.94 |

**Supplementary Table 5. Univariable Analysis for Risk Factors for Recurrence Free Survival in Patients who received Neoadjuvant Therapy**

|  | Univariable | |
| --- | --- | --- |
|  | HR [95% CI] | p-value |
| Age >65 (Ref: Age <65) | 0.43 [0.16 - 1.16] | 0.10 |
| Male Sex (Ref: Female) | 0.73 [0.32 - 1.71] | 0.47 |
| Stage II (Ref: Stage I) | **4.29 [1.26 - 14.57]** | **0.02** |
| Positive Fungal Culture (Ref: Negative Culture) | 1.20 [0.52 - 2.77] | 0.67 |
| Positive Bacterial Culture (Ref: Negative Culture) | 0.48 [0.16 - 1.45] | 0.20 |
| *Enterococcus spp.* | 0.79 [0.34 - 1.81] | 0.57 |
| *Streptococcus spp.* | 0.53 [0.22 - 1.26] | 0.15 |
| *Klebsiella spp.* | 0.56 [0.23 - 1.37] | 0.20 |
| *Enterobacter spp.* | 0.68 [0.25 - 1.86] | 0.46 |
| *Clostridium spp.* | 1.29e-8 [0 - $\infty$] | 1.00 |
| *Escherichia spp.* | 1.28 [0.47 - 3.50] | 0.64 |
| *Prevotella spp.* | 0.65 [0.24 - 1.75] | 0.39 |
| Received Anti-Fungal Drug | 1.02 [0.34 - 3.01] | 0.97 |

**Supplementary Table 6. Univariable Analysis for Risk Factors for Overall Survival in Patients who received Upfront Resection**

|  | Univariable | |
| --- | --- | --- |
|  | HR [95% CI] | p-value |
| Age >65 (Ref: Age <65) | 1.87 [0.53 - 6.61] | 0.33 |
| Male Sex (Ref: Female) | 0.65 [0.16 - 2.72] | 0.56 |
| ASA Class (Ref: Class 2) |  |  |
| Class 3 | 0.98 [0.28 - 3.41] | 0.97 |
| Stage II (Ref: Stage I) | 1.38e-8 [0 - $\infty$] | 1.00 |
| Positive Fungal Culture (Ref: Negative Culture) | 1.34 [0.27 - 6.77] | 0.72 |
| Positive Bacterial Culture (Ref: Negative Culture) | 1.41 [0.29 - 6.84] | 0.67 |
| *Enterococcus spp.* | 0.68 [0.20 - 2.38] | 0.55 |
| *Streptococcus spp.* | 1.57 [0.44 - 5.57] | 0.48 |
| *Klebsiella spp.* | 1.72 [0.41 - 7.26] | 0.46 |
| *Enterobacter spp.* | 0.32 [0.04 - 2.58] | 0.28 |
| *Escherichia spp.* | 1.07 [0.13 - 8.90] | 0.95 |
| *Prevotella spp.* | 1.18e-8 [0 - $\infty$] | 1.00 |
| *Clostridium spp.* | 1.09 [0.28 - 4.25] | 0.90 |
| Received Anti-Fungal Drug | 2.45 [0.58 - 10.29] | 0.22 |
| Any Complication Occurrence (Ref: No Complication) | 1.93 [0.54 - 6.90] | 0.31 |

**Supplementary Table 7. Univariable Analysis for Risk Factors for Recurrence Free Survival in Patients who received Upfront Resection**

|  | Univariable | |
| --- | --- | --- |
|  | HR [95% CI] | p-value |
| Age >65 (Ref: Age <65) | 0.66 [0.13 - 3.38] | 0.62 |
| Male Sex (Ref: Female) | 2.02 [0.48 - 8.48] | 0.34 |
| Stage II (Ref: Stage I) | 7.43e8 [0 - $\infty$] | 1.00 |
| Positive Fungal Culture (Ref: Negative Culture) | 1.46 [0.29 - 7.34] | 0.65 |
| Positive Bacterial Culture (Ref: Negative Culture) | 0.90 [0.18 - 4.52] | 0.90 |
| *Enterococcus spp.* | 0.39 [0.09 - 1.64] | 0.20 |
| *Streptococcus spp.* | 2.15 [0.53 - 8.71] | 0.28 |
| *Klebsiella spp.* | 0.40 [0.05 - 3.32] | 0.40 |
| *Enterobacter spp.* | 0.63 [0.13 - 3.16] | 0.58 |
| *Clostridium spp.* | 2.04 [0.47 - 8.80] | 0.34 |
| *Escherichia spp.* | 1.01 [0.12 - 8.40] | 0.99 |
| *Prevotella spp.* | 1.22 [0.14 - 10.34] | 0.86 |
| Received Anti-Fungal Drug | 1.27 [0.26 - 6.32] | 0.77 |

# Supplementary Figures


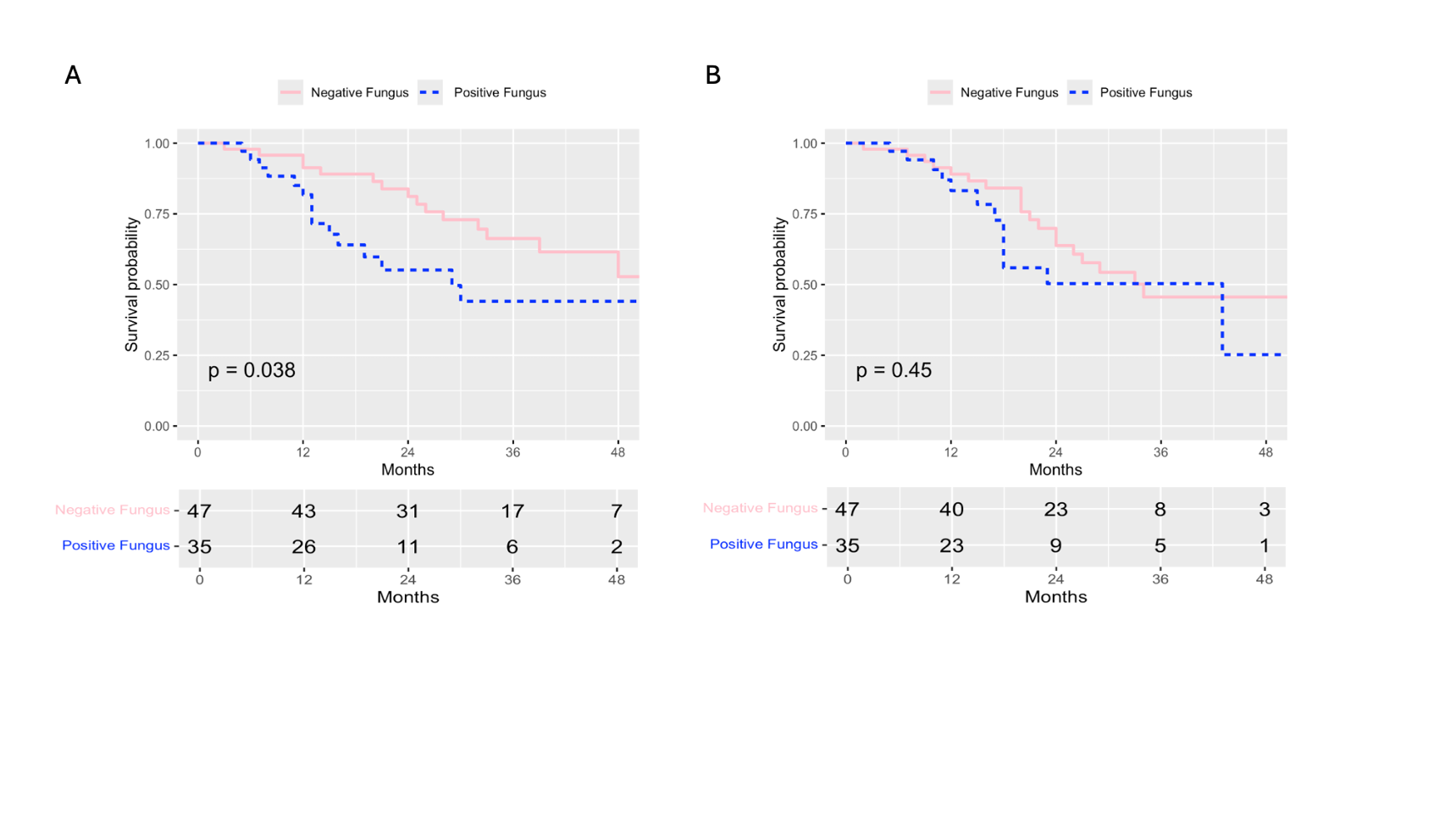


**Supplementary Figure 1.** Kaplan Meier Curves for (A) OS and (B) RFS in patients with positive and negative fungal bile cultures. Kaplan Meier Curves were compared with log rank test for alpha significance. (A) Median OS was 29 months (95% CI: 16 – Not reached) for positive bile fungus vs. 53 months (95% CI: 39 – Not reached) for negative bile fungus, p=0.038. N=47 for negative fungus with 19 death events. N=35 for positive fungus with 15 death events. (B) Median RFS was 43 months (95% CI: 18 – Not reached) for positive bile fungus vs. 34 months (95% CI: 26 – Not reached) for negative bile fungus, p=0.45. N=47 for negative fungus with 19 recurrence events. N=35 for positive fungus with 12 recurrence events.
